# Bis(trimethyl­ammonium) naphthalene-1,5-disulfonate

**DOI:** 10.1107/S1600536811052718

**Published:** 2011-12-10

**Authors:** Yu Jin

**Affiliations:** a Ordered Matter Science Research Center, Southeast University, Nanjing 211189, People’s Republic of China

## Abstract

The asymmetric unit of the title compound, 2C_3_H_10_N^+^·C_10_H_6_S_2_O_6_^2−^, contains a half-anion, which is completed by inversion symmetry, and one cation. The cations and anions are associated *via* strong N—H⋯O(sulfonate) hydrogen-bonding inter­actions, forming cation–anion–cation groups. Secondary inter­actions such as C—H(ammonium)⋯O(sulf­on­ate) and van der Waals inter­actions link the cations and anions together in a three-dimensional crystal structure, with zigzag rows of cations lying between layers of anions.

## Related literature

The title compound was  investigated as part of our search for simple ferroelectric compounds. For general background to ferroelectric metal-organic frameworks, see: Ye *et al.* (2006 ▶); Zhang *et al.* (2008 ▶, 2009 ▶, 2010 ▶); Fu *et al.* (2009 ▶). For a related structure, see: Wang & Yang (2011 ▶).
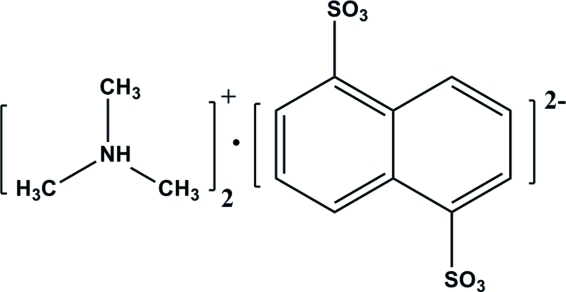


## Experimental

### 

#### Crystal data

2C_3_H_10_N^+^·C_10_H_6_O_6_S_2_^2−^
*M* *_r_* = 406.51
Monoclinic, 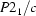

*a* = 8.3428 (17) Å
*b* = 10.502 (2) Å
*c* = 11.742 (2) Å
β = 105.81 (3)°
*V* = 989.8 (3) Å^3^
*Z* = 2
Mo *K*α radiation
μ = 0.30 mm^−1^
*T* = 293 K
0.3 × 0.3 × 0.2 mm

#### Data collection

Rigaku Mercury CCD diffractometer
Absorption correction: multi-scan (*CrystalClear*; Rigaku, 2005 ▶) *T*_min_ = 0.489, *T*_max_ = 1.000
10031 measured reflections
2265 independent reflections
2016 reflections with *I* > 2σ(*I*)
*R*_int_ = 0.036

#### Refinement

*R*[*F* ^2^ > 2σ(*F* ^2^)] = 0.041
*wR*(*F* ^2^) = 0.109
*S* = 1.10
2265 reflections
119 parameters
H-atom parameters constrained
Δρ_max_ = 0.28 e Å^−3^
Δρ_min_ = −0.36 e Å^−3^

### 

Data collection: *CrystalClear* (Rigaku, 2005 ▶); cell refinement: *CrystalClear*; data reduction: *CrystalClear*; program(s) used to solve structure: *SHELXS97* (Sheldrick, 2008 ▶); program(s) used to refine structure: *SHELXL97* (Sheldrick, 2008 ▶); molecular graphics: *SHELXTL* (Sheldrick, 2008 ▶); software used to prepare material for publication: *SHELXL97*.

## Supplementary Material

Crystal structure: contains datablock(s) I, global. DOI: 10.1107/S1600536811052718/bh2404sup1.cif

Structure factors: contains datablock(s) I. DOI: 10.1107/S1600536811052718/bh2404Isup2.hkl

Supplementary material file. DOI: 10.1107/S1600536811052718/bh2404Isup3.cml

Additional supplementary materials:  crystallographic information; 3D view; checkCIF report

## Figures and Tables

**Table 1:** Hydrogen-bond geometry (Å, °)

*D*—H⋯*A*	*D*—H	H⋯*A*	*D*⋯*A*	*D*—H⋯*A*
N1—H1*E*⋯O2^i^	0.91	1.81	2.718 (2)	173
C1—H1*C*⋯O1	0.96	2.43	3.372 (3)	166
C2—H2*B*⋯O3^ii^	0.96	2.31	3.232 (4)	162

Symmetry codes: (i) 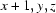; (ii) 
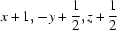
.

**Fig. 1.**
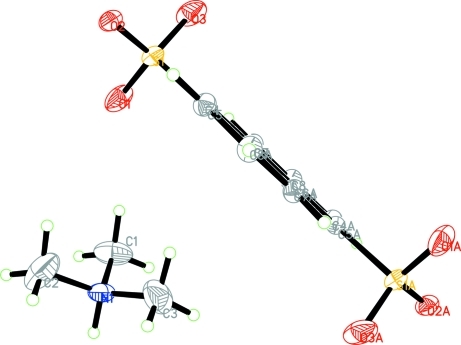
The molecular structure of the title compound with displacement ellipsoids drawn at the 30% probability level.

**Fig. 2.**
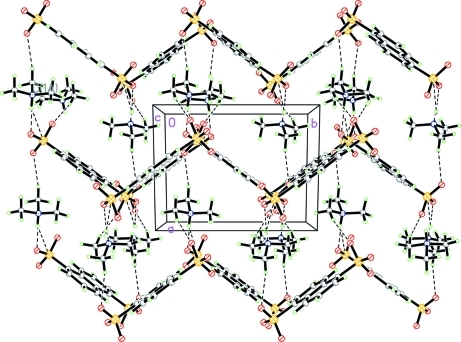
The crystal structure of the title compound viewed along the c axis. Intermolecular interactions are shown as dashed lines.

## supplementary crystallographic information

### Comment

Ferroelectric compounds have displayed a variety of technical applications, such as ferroelectric random access memories, infrared detectors, piezoelectric sensors, nonlinear optical devices, as a result of their excellent ferroelectric, piezoelectric, pyroelectric, and optical properties. Most of the new ferroelectric metal-organic compounds consistent with the necessary requirements for ferroelectric properties have been explored. However, the conditions required in these systems, such as a phase transition, a good electric hysteresis loop and electric domain, and a dielectric anomaly, often disappear (Zhang *et al.*, 2009). Hence, pure organic compounds can be of great potential and can probably make up for the drawbacks found in ferroelectric metal-organic compounds. Reversible structural phase transition remains one of the prominent properties for ferroelectrics. For a small part of these compounds, the components can be arranged in a disordered fashion at a relative high temperature and in an ordered fashion at a relative low temperature. The transition from the disordered arrangement to the ordered one gives rise to sharp change in the physical properties of the compound (Fu *et al.*, 2009; Zhang *et al.*, 2008, 2010; Ye *et al.*, 2006). As part of our search for simple ferroelectric compounds, we have investigated the title compound and report now its room temperature crystal structure.

The centrosymmetric anion and one cation are shown in Fig. 1 with the hydrogen bonds listed in Table 1. The existence of numerous hydrogen-bonding interactions helps to make the substance more stable, and thus forms a three-dimensional layered structure. The interactions tie the cations and anions together in sheets with zigzag rows of cations lying between layers of anions (Fig. 2). There are only van der Waals interactions between layers. The reported structure is similar to that of a related naphthalene-1,5-disulfonate salt (Wang & Yang, 2011).

### Experimental

(C_3_H_10_N^+^)_2_(C_10_H_6_S_2_O_6_^2-^) was formed from a mixture of N(CH_3_)_3_ (8 mL), C_10_H_8_O_6_S_2_ (288.28 mg, 1.00 mmol), and distilled water (10 ml), which was stirred for few minutes at room temperature, giving a clear transparent solution. After evaporation over few days, block-shaped colorless crystals suitable for X-ray diffraction were obtained in about 82% yield, filtered and washed with distilled water.

### Refinement

H atoms bound to carbon and nitrogen were placed at idealized positions [C—H = 0.93–0.96 Å, N—H = 0.91 Å] and allowed to ride on their parent atoms with *U*_iso_ fixed at 1.2 *U*_eq_(C,N).

### Crystal data

**Table:**

2C_3_H_10_N^+^·C_10_H_6_O_6_S_2_^2^^−^	*F*(000) = 432
*M**_r_* = 406.51	*D*_x_ = 1.364 Mg m^−^^3^
Monoclinic, *P*2_1_/*c*	Mo *K*α radiation, λ = 0.71073 Å
Hall symbol: -P 2ybc	Cell parameters from 3450 reflections
*a* = 8.3428 (17) Å	θ = 6.2–55.3°
*b* = 10.502 (2) Å	µ = 0.30 mm^−^^1^
*c* = 11.742 (2) Å	*T* = 293 K
β = 105.81 (3)°	Block, colourless
*V* = 989.8 (3) Å^3^	0.3 × 0.3 × 0.2 mm
*Z* = 2	

### Data collection

**Table:**

Rigaku Mercury CCD diffractometer	2265 independent reflections
Radiation source: fine-focus sealed tube	2016 reflections with *I* > 2σ(*I*)
graphite	*R*_int_ = 0.036
ω scans	θ_max_ = 27.5°, θ_min_ = 3.2°
Absorption correction: multi-scan (*CrystalClear*; Rigaku, 2005)	*h* = −10→10
*T*_min_ = 0.489, *T*_max_ = 1.000	*k* = −13→13
10031 measured reflections	*l* = −15→15

### Refinement

**Table:**

Refinement on *F*^2^	Secondary atom site location: difference Fourier map
Least-squares matrix: full	Hydrogen site location: inferred from neighbouring sites
*R*[*F*^2^ > 2σ(*F*^2^)] = 0.041	H-atom parameters constrained
*wR*(*F*^2^) = 0.109	*w* = 1/[σ^2^(*F*_o_^2^) + (0.0505*P*)^2^ + 0.3652*P*] where *P* = (*F*_o_^2^ + 2*F*_c_^2^)/3
*S* = 1.10	(Δ/σ)_max_ < 0.001
2265 reflections	Δρ_max_ = 0.28 e Å^−^^3^
119 parameters	Δρ_min_ = −0.36 e Å^−^^3^
0 restraints	Extinction correction: *SHELXL97* (Sheldrick, 2008), Fc^*^=kFc[1+0.001xFc^2^λ^3^/sin(2θ)]^-1/4^
0 constraints	Extinction coefficient: 0.055 (4)
Primary atom site location: structure-invariant direct methods	

### Fractional atomic coordinates and isotropic or equivalent isotropic displacement parameters (Å^2^)

**Table:**

	*x*	*y*	*z*	*U*_iso_*/*U*_eq_	
C1	0.7732 (3)	0.1761 (3)	0.4624 (2)	0.0783 (8)	
H1B	0.7949	0.2389	0.4090	0.117*	
H1C	0.6593	0.1826	0.4647	0.117*	
H1D	0.7929	0.0927	0.4357	0.117*	
C2	0.8549 (4)	0.1033 (3)	0.6651 (3)	0.0926 (10)	
H2B	0.9280	0.1194	0.7423	0.139*	
H2C	0.8760	0.0197	0.6395	0.139*	
H2D	0.7413	0.1087	0.6683	0.139*	
C3	0.8748 (4)	0.3266 (3)	0.6265 (3)	0.0824 (9)	
H3B	0.9491	0.3339	0.7046	0.124*	
H3C	0.7629	0.3436	0.6293	0.124*	
H3D	0.9061	0.3868	0.5749	0.124*	
C4	0.4055 (2)	0.39604 (16)	0.66437 (14)	0.0336 (4)	
H4A	0.3567	0.3452	0.7105	0.040*	
C5	0.37869 (18)	0.37009 (14)	0.54673 (13)	0.0279 (3)	
C6	0.44909 (18)	0.44781 (14)	0.47321 (13)	0.0258 (3)	
C7	0.4218 (2)	0.42584 (15)	0.35041 (14)	0.0315 (4)	
H7A	0.3535	0.3589	0.3146	0.038*	
C8	0.4940 (2)	0.50113 (16)	0.28418 (14)	0.0353 (4)	
H8A	0.4764	0.4842	0.2040	0.042*	
N1	0.88425 (18)	0.19790 (16)	0.58184 (15)	0.0444 (4)	
H1E	0.9904	0.1864	0.5772	0.053*	
O1	0.3624 (2)	0.15499 (14)	0.43979 (16)	0.0635 (5)	
O2	0.21016 (15)	0.17580 (12)	0.58538 (12)	0.0406 (3)	
O3	0.11286 (19)	0.28226 (17)	0.39993 (14)	0.0646 (5)	
S1	0.25686 (5)	0.23499 (4)	0.48711 (4)	0.03500 (17)	

### Atomic displacement parameters (Å^2^)

**Table:**

	*U*^11^	*U*^22^	*U*^33^	*U*^12^	*U*^13^	*U*^23^
C1	0.0441 (12)	0.127 (3)	0.0569 (15)	−0.0063 (14)	0.0017 (11)	−0.0125 (16)
C2	0.112 (2)	0.086 (2)	0.0729 (18)	−0.0519 (19)	0.0134 (17)	0.0023 (15)
C3	0.0762 (18)	0.0563 (15)	0.104 (2)	0.0190 (13)	0.0066 (16)	−0.0161 (14)
C4	0.0363 (8)	0.0363 (8)	0.0301 (8)	−0.0014 (7)	0.0123 (7)	0.0050 (6)
C5	0.0247 (7)	0.0274 (7)	0.0310 (8)	0.0002 (6)	0.0068 (6)	0.0011 (6)
C6	0.0256 (7)	0.0249 (7)	0.0265 (7)	0.0029 (6)	0.0064 (6)	0.0005 (6)
C7	0.0347 (8)	0.0298 (8)	0.0285 (8)	−0.0014 (6)	0.0061 (6)	−0.0041 (6)
C8	0.0423 (9)	0.0385 (9)	0.0254 (7)	−0.0014 (7)	0.0100 (7)	−0.0011 (6)
N1	0.0267 (7)	0.0521 (10)	0.0534 (10)	−0.0035 (6)	0.0092 (7)	−0.0075 (8)
O1	0.0751 (11)	0.0409 (8)	0.0925 (12)	−0.0193 (7)	0.0537 (10)	−0.0242 (8)
O2	0.0332 (6)	0.0420 (7)	0.0471 (7)	−0.0069 (5)	0.0118 (5)	0.0085 (5)
O3	0.0455 (8)	0.0811 (11)	0.0533 (9)	−0.0226 (8)	−0.0101 (7)	0.0189 (8)
S1	0.0322 (2)	0.0350 (3)	0.0379 (3)	−0.00957 (16)	0.00984 (17)	−0.00121 (16)

### Geometric parameters (Å, °)

**Table:**

C1—N1	1.473 (3)	C4—H4A	0.9300
C1—H1B	0.9600	C5—C6	1.426 (2)
C1—H1C	0.9600	C5—S1	1.7744 (16)
C1—H1D	0.9600	C6—C7	1.416 (2)
C2—N1	1.460 (3)	C6—C6^i^	1.424 (3)
C2—H2B	0.9600	C7—C8	1.359 (2)
C2—H2C	0.9600	C7—H7A	0.9300
C2—H2D	0.9600	C8—C4^i^	1.400 (2)
C3—N1	1.459 (3)	C8—H8A	0.9300
C3—H3B	0.9600	N1—H1E	0.9100
C3—H3C	0.9600	O1—S1	1.4338 (15)
C3—H3D	0.9600	O2—S1	1.4541 (13)
C4—C5	1.365 (2)	O3—S1	1.4379 (16)
C4—C8^i^	1.400 (2)		
			
N1—C1—H1B	109.5	C6—C5—S1	120.33 (11)
N1—C1—H1C	109.5	C7—C6—C6^i^	119.11 (17)
H1B—C1—H1C	109.5	C7—C6—C5	123.01 (14)
N1—C1—H1D	109.5	C6^i^—C6—C5	117.88 (16)
H1B—C1—H1D	109.5	C8—C7—C6	120.95 (15)
H1C—C1—H1D	109.5	C8—C7—H7A	119.5
N1—C2—H2B	109.5	C6—C7—H7A	119.5
N1—C2—H2C	109.5	C7—C8—C4^i^	120.58 (15)
H2B—C2—H2C	109.5	C7—C8—H8A	119.7
N1—C2—H2D	109.5	C4^i^—C8—H8A	119.7
H2B—C2—H2D	109.5	C3—N1—C2	110.7 (2)
H2C—C2—H2D	109.5	C3—N1—C1	113.8 (2)
N1—C3—H3B	109.5	C2—N1—C1	110.8 (2)
N1—C3—H3C	109.5	C3—N1—H1E	107.0
H3B—C3—H3C	109.5	C2—N1—H1E	107.0
N1—C3—H3D	109.5	C1—N1—H1E	107.0
H3B—C3—H3D	109.5	O1—S1—O3	114.11 (11)
H3C—C3—H3D	109.5	O1—S1—O2	112.49 (9)
C5—C4—C8^i^	120.29 (15)	O3—S1—O2	111.10 (8)
C5—C4—H4A	119.9	O1—S1—C5	105.98 (8)
C8^i^—C4—H4A	119.9	O3—S1—C5	106.41 (9)
C4—C5—C6	121.16 (14)	O2—S1—C5	106.10 (8)
C4—C5—S1	118.50 (12)		

Symmetry codes: (i) −*x*+1, −*y*+1, −*z*+1.

### Hydrogen-bond geometry (Å, °)

**Table:**

*D*—H···*A*	*D*—H	H···*A*	*D*···*A*	*D*—H···*A*
N1—H1E···O2^ii^	0.91	1.81	2.718 (2)	173.
C1—H1C···O1	0.96	2.43	3.372 (3)	166
C2—H2B···O3^iii^	0.96	2.31	3.232 (4)	162
N1—H1E···S1^ii^	0.91	2.76	3.5967 (18)	154.

Symmetry codes: (ii) *x*+1, *y*, *z*; (iii) *x*+1, −*y*+1/2, *z*+1/2.

## References

[bb1] Fu, D.-W., Ge, J.-Z., Dai, J., Ye, H.-Y. & Qu, Z.-R. (2009). *Inorg. Chem. Commun.* **12**, 994–997.

[bb2] Rigaku (2005). *CrystalClear* Rigaku Corporation, Tokyo, Japan.

[bb3] Sheldrick, G. M. (2008). *Acta Cryst.* A**64**, 112–122. 10.1107/S0108767307043930 18156677

[bb4] Wang, C. & Yang, S. L. (2011). *Acta Cryst.* E**67**, o1847. 10.1107/S1600536811023269 PMC3151963 21837212

[bb5] Ye, Q., Song, Y.-M., Wang, G.-X., Chen, K., Fu, D.-W., Hong Chan, P. W., Zhu, J.-S., Huang, S. D. & Xiong, R.-G. (2006). *J. Am. Chem. Soc.* **128**, 6554–6555. 10.1021/ja060856p 16704244

[bb6] Zhang, W., Chen, L.-Z., Xiong, R.-G., Nakamura, T. & Huang, S. D. (2009). *J. Am. Chem. Soc.* **131**, 12544–12545. 10.1021/ja905399x 19685869

[bb7] Zhang, W., Xiong, R.-G. & Huang, S. D. (2008). *J. Am. Chem. Soc.* **130**, 10468–10469. 10.1021/ja803021v 18636707

[bb8] Zhang, W., Ye, H.-Y., Cai, H.-L., Ge, J.-Z., Xiong, R.-G. & Huang, S. D. (2010). *J. Am. Chem. Soc.* **132**, 7300–7302. 10.1021/ja102573h 20459097

